# Establishment of a DNA-free genome editing and protoplast regeneration method in cultivated tomato (*Solanum lycopersicum*)

**DOI:** 10.1007/s00299-022-02893-8

**Published:** 2022-06-30

**Authors:** Ying Liu, Mariette Andersson, Antonio Granell, Teodoro Cardi, Per Hofvander, Alessandro Nicolia

**Affiliations:** 1 Department of Plant Breeding, Swedish University of Agricultural Sciences, P.O. Box 190, 23422 Lomma, Sweden; 2 Instituto de Biología Molecular Y Celular de Plantas, CSIC-Universidad Politécnica de Valencia, 46022 Valencia, Spain; 3 Council for Agricultural Research and Economics, Research Centre for Vegetable and Ornamental Crops, Via Cavalleggeri 25, 84098 Pontecagnano, Italy; 4 Institute of Biosciences and Bioresources, CNR-IBBR, via Università 133, 80055 Portici, Italy

**Keywords:** *Solanum lycopersicum*, Mesophyll protoplast regeneration, CRISPR/Cas9, Ribonucleoprotein, *SP* and *SP5G* genes

## Abstract

**Key message:**

**We have established a DNA-free genome editing method via ribonucleoprotein-based CRISPR/Cas9 in cultivated tomato and obtained mutant plants regenerated from transfected protoplasts with a high mutation rate.**

**Abstract:**

The application of genome editing as a research and breeding method has provided many possibilities to improve traits in many crops in recent years. In cultivated tomato (*Solanum lycopersicum*), so far only stable *Agrobacterium*-mediated transformation carrying CRISPR/Cas9 reagents has been established. Shoot regeneration from transfected protoplasts is the major bottleneck in the application of DNA-free genome editing via ribonucleoprotein-based CRISPR/Cas9 method in cultivated tomato. In this study, we report the implementation of a transgene-free breeding method for cultivated tomato by CRISPR/Cas9 technology, including the optimization of protoplast isolation and overcoming the obstacle in shoot regeneration from transfected protoplasts. We have identified that the shoot regeneration medium containing 0.1 mg/L IAA and 0.75 mg/L zeatin was the best hormone combination with a regeneration rate of up to 21.3%. We have successfully obtained regenerated plants with a high mutation rate four months after protoplast isolation and transfection. Out of 110 regenerated M_0_ plants obtained, 35 (31.8%) were mutated targeting both *SP* and *SP5G* genes simultaneously and the editing efficiency was up to 60% in at least one allele in either *SP* or *SP5G* genes.

**Supplementary Information:**

The online version contains supplementary material available at 10.1007/s00299-022-02893-8.

## Introduction

Variations of CRISPR/Cas9 technology have been applied for genome editing in recent years (Gao 2021). This technology has surpassed the other genome editing tools, such as zinc finger nucleases (ZFNs) and transcription activator-like effector nucleases (TALENs), as it is more easily and cheaply customized and yields high mutation efficiency in some species (Lowder et al. 2015). So far, successful applications using CRISPR/Cas9 have been reported in model plants, such as *Arabidopsis thaliana* (Li et al. 2013; Yan et al. 2015) and *Nicotiana benthamiana* (Li et al. 2013; Nekrasov et al. 2013), and many commercial crops, such as potato (Wang et al. 2015; Andersson et al. 2017), wheat (Upadhyay et al. 2013; Zhang et al. 2016), rice (Jiang et al. 2013; Zhou et al. 2014), maize (Liang et al. 2014; Char et al. 2016), tomato (Brooks et al. 2014; Ito et al. 2015) and many others (Gao 2021).

In general, CRISPR/Cas9 reagents, usually as DNA plasmids, can be delivered to cell-wall-free protoplasts by polyethylene glycol (PEG) transformation, or to plant tissues by stable *Agrobacterium*-mediated transformation, or by other means, such as particle bombardment and electroporation (Chen et al. 2019). To avoid foreign DNA integrated into plant cells, Woo et al. (2015) were the first to develop the delivery of Ribonucleoprotein (RNP) complexes into plant protoplasts using in vitro preassembled complexes of purified Cas9 protein and guide RNA (gRNA) in *Arabidopsis*, tobacco, lettuce and rice. Subsequently, studies on the application of RNPs for genome editing in plant species have been reported in crop plants, such as maize (Svitashev et al. 2016), wheat (Liang et al. 2017), potato (Andersson et al. 2018) and canola (Sidorov et al. 2021).

Tomato (*Solanum lycopersicum*) is an important commercial agricultural crop which is extensively cultivated all over the world as well as being a model plant used in scientific research due to its simple diploid genetics (2n = 2x = 24) and short life cycle (Ito et al. 2015). It has been demonstrated that the CRISPR/Cas9 technology can be used to generate mutated tomato plants for crop improvement such as improved disease resistance (Ito et al. 2015; Pan et al. 2016). Hitherto, most reports about CRISPR/Cas9 applied to tomato were based on stable *Agrobacterium tumefaciens*-mediated transformation and usually the homozygous mutation rate was low and segregation in the next generation needed to eliminate foreign DNA integrated into the plant genome. An attractive alternative technology would be the use of RNPs to deliver the CRISPR/Cas9 reagents into protoplasts, resulting in transgene-free plants. Although a high editing efficiency has been reported on tomato calli from transfected protoplasts of cultivated tomato, the shoot regeneration from RNP-transfected protoplasts is a bottleneck (Nicolia et al. 2021a). Very recently, (Lin et al. 2022) reported the successful protoplast regeneration of wild tomato (*Solanum peruvianum*) harboring CRISRP/Cas9 mutations, with a mutation rate varying from 8.3% to 63.6%. However, for cultivated tomato, there are only a few old reports published on plant regeneration from unedited protoplasts (Morgan and Cocking 1982; Sakata et al. 1987; Tan et al. 1987). Thus, establishing a protocol with high editing efficiency and regeneration rate in cultivated tomato would be beneficial regarding genetic studies as well as for breeding purposes. From this aspect, such a protocol could also be further adapted to wild relatives of the tomato, that represent a precious source of variability (e.g., *Solanum pennellii*, *Solanum pimpinellifolium*), to speed up programs of “de novo” domestication and/or introgression (Li et al. 2018).

The vegetative-to-reproductive phases in tomato are altered in the sympodial shoots and the switch between those two phases is controlled by the flowering repressor gene *SELF PRUNING* (*SP*). Genetic variation and mutations in this gene yields tomato genotypes that are classified into two categories: 'determinate' and 'indeterminate' varieties due to different growth habits (Pnueli et al. 1998; Carmel-Goren et al. 2003). Another flowering repressor *SELF PRUNING 5G* (*SP5G*), which is a paralog of the *SP* gene, is mainly responsible for flower repression in primary and canonical axillary shoots (Soyk et al. 2017). Tomato plants with mutation in either the *SP* gene or both the *SP* and *SP5G* genes showed the determinate phenotype, which resulted in acceleration of flowering, short internodes, bushy appearance and rapid life cycling (Soyk et al. 2017; Kwon et al. 2020). Those mutated plants would be suitable for urban vertical farming and greenhouse cultivation since the agricultural productivity can be increased due to their fast growth habit and compact size, especially in a confined environment. It is also beneficial for open field cultivation in that they grow as small bushes that need less attention compared to indeterminate varieties needing support. Besides, all fruits from determinate cultivars usually ripen in a short period from simultaneous flowering, which is beneficial for facilitating mechanical harvest.

In this study, we have successfully regenerated plants from cultivated tomato transfected protoplasts within four months after transfection. Furthermore, the regenerated plants have a high editing rate when targeting both *SP* and *SP5G* genes simultaneously. Hence, we have improved the process of tomato protoplast isolation and solved the challenge of shoot regeneration from RNP-transfected protoplasts using CRISPR/Cas9 technology.

## Materials and methods

### Plant material and in vitro culture conditions

Seeds of tomato (*S. lycopersicum*) cultivars (cvs) Red Setter, Ailsa Craig, M82 and Moneymaker were used in this study. Seeds were surface sterilized by washing with 70% ethanol for 5 min, followed by 15% (w/v) calcium hypochlorite (CaCl_2_O_2_) for 3 min and then rinsed 5 times with sterile distilled water. Sterilized seeds were placed in Plante Containers (Sakata Ornamentals Europe A/S, Denmark) with germination medium containing 0.2 mg/L Indole Acetic acid (IAA), 15 g/L sucrose, 8 g/L phyto agar, and half-strength Murashige & Skoog (MS) with vitamins (Duchefa Biochemie M0222, Haarlem, Netherlands) (2.2 g/L) with additional 0.2 mg/L Thiamine and 50 mg/L Myo-Inositol at pH 5.9.

In vitro culture mentioned in this study was carried out in a controlled chamber at a temperature of 24 °C/18 °C (light/dark), under a photoperiod of 16 h at 120–140 μE m^−2^ s^−1^ light and 8 h dark.

### Protoplast isolation, transfection and callus induction in liquid medium

Protoplast isolation, transfection and early callus induction in liquid medium were done as previously described by Nicolia et al. (2021b) with some modifications to improve the yield of isolated protoplasts and regeneration. The components of Medium C, E, F, wash solution, PEG solution, alginate solution and transient expression solution mentioned below can be found in Nicolia et al. (2021b).

In brief, the modifications in protoplast isolation were as follows: preconditioning treatment of in vitro cultured seedlings prior to protoplast isolation was done by placing the Plante Containers in a fridge (4 °C) in darkness one day before isolation. Cotyledons and first true leaves at different ages (14, 17 and 21 d) were used for protoplast isolation. The leaf tissues were sliced and treated with enzyme solution (medium C) at different temperatures (15 and 25 °C) and different time durations of enzyme digestion (14 and 16 h). Protoplasts were collected after centrifugation and the protoplast yield was quantified immediately after isolation by a hemocytometer (FuchsRosenthal 0.2 mm chamber, Horsham, UK) under microscope. The optimization of protoplast isolation was carried out with two cvs (Red Setter and Ailsa Craig) and optimized conditions were confirmed in all four cvs.

Freshly isolated protoplasts were transfected via PEG mediated delivery of RNPs. For each transfection, two different RNP complexes were assembled by mixing two 0.1 nmol synthetic sgRNAs (Synthego) with 10 µg TrueCut™ Cas9 v.2 (Thermo Fisher, Waltham, USA) in a 15 ml tube at room temperature for 15 min. The sgRNA was used as one synthetically produced component including the 20 bp target and an 80-mer SpCas9 scaffold from the suppliers’ standard products. Then, 100 µl of protoplast suspension (1.0 × 10^6^ protoplasts/ml) was added to the same tube and gently mixed before and after adding 120 µl 25% (w/v) PEG solution. The transfection was stopped after 3 min by 5 ml wash solution. Two control experiments, one with and one without PEG solution were also conducted. For estimation of transfection efficiency, protoplasts were transfected by replacing RNP with 20 µg plasmid vector expressing Green Fluorescent Protein (GFP) (pCW498-35S-GFiP-OcsT) and incubated with transient expression solution at room temperature in darkness. After 24 h, the expression of GFP signal was detected under a confocal microscope (Zeiss LSM 880 Airyscan confocal laser scanning microscope, Oberkochen, Germany).

After transfection, protoplasts were embedded in alginate and incubated in Medium E at 25 °C in darkness for 5 d where after the light was gradually increased by replacing the aluminum foil with a white paper sheet under the light intensity at ca. 10 μE m^−2^ s^−1^. After two weeks, Medium E was replaced by Medium F and calli were exposed to full light with fresh Medium F changed every week.

### Shoot and root regeneration on solid medium

After two weeks of incubation in Medium F, calli of 1–3 mm in size were released from the alginate using forceps and transferred directly to solid media for further shoot regeneration. Different solid media were designed to test their respective potential for tomato protoplast shoot regeneration. The composition of each medium is listed in Supp. Table 1. Solid shoot media were renewed every two weeks until shoots were regenerated. The number of regenerated shoots was evaluated continuously on different regeneration media until six months after protoplast isolation.

Individual regenerated shoots were excised from calli and moved to root regeneration medium containing 4.405 g/L MS medium with vitamins, 30 g/L sucrose and 6 g/L phyto agar at pH 5.8 in Plante Containers. Regenerated plants with roots were moved to soil for subsequent seed production and phenotypic observation.

### Identification of *SP* and *SP5G* genes and sgRNA design

Genomic DNA was extracted from tomato leaf tissue of the four tomato cultivars using the GeneJet Plant Genomic DNA Purification Mini Kit (Thermo Fisher Scientific, Waltham USA) for amplification of the target regions in *SP* and *SP5G* genes. For each gene, two pairs of primers were designed based on the sequence of Solyc06g074350 (*SP*) and Solyc05g053850 (*SP5G*) (https://solgenomics.net/). Amplification of the target regions was conducted in a total reaction of 10 µl containing 5X Phusion HF buffer, 0.2 mM dNTPS, 0.15 µM primers, 0.02 U/µl Phusion DNA polymerase and 1 µl of extracted gDNA. PCR was conducted as follows: 98 ℃ for 1 min, 35 cycles of 98 °C 10 s, 59 °C 15 s, 72 °C 15 s and a final extension of 72 °C for 10 min. PCR products were cloned using the CloneJET PCR cloning Kit (Thermo Fisher Scientific, Waltham USA) and six random clones from each amplicon were selected for Sanger Sequencing (Eurofins). sgRNAs were designed to target all alleles in the four tomato cvs. according to the Sanger Sequencing using CRISPR RGEN Cas-Designer (Bae et al. 2014) and CRISPOR (Concordet and Haeussler 2018). All primers and sgRNAs used in this study are summarized in Supp. Table 2.

### Genotyping of *SP* and *SP5G* mutants

Initial screening of mutations was performed with High Resolution Fragment Analysis (HRFA) according to Andersson et al. (2017). Genomic DNA was extracted from single young leaf tissue from each in vitro regenerated plant using GeneJet Plant Genomic DNA Purification Mini Kit. Multiplexing PCR was applied to amplify the regions covering target sites of both *SP* and *SP5G* genes simultaneously with forward primers labeled with FAM and HEX fluorescent dye (Thermo Fisher Scientific, Waltham USA), respectively. Labeled amplicons were analyzed in a 3500 Genetic Analyzer (Applied Biosystems) and the size of fragments was determined with GeneMarker Software (SoftGenetics, Pennsylvania, USA) compared with the size of wild type amplicons. Sanger Sequencing was conducted for further characterization of mutations using unlabeled primers.

### Statistical analysis

To evaluate the effect of different media on shoot regeneration rate, the number of shoots on each medium was recorded until six months after protoplast isolation. On the representative media (Medium TRS-a, b and c), the mean regeneration rate of each treatment was calculated with three replicates on individual culture dishes, containing ca. 40–60 calli per replicate. For other shoot regeneration media, the regeneration rate of each treatment was calculated based on one replicate containing 50 calli. Data were analyzed with one-way ANOVA and Tukey’s test using software IBM SPSS Statistics version 27.

## Results

### Improvement on protoplast isolation

**Fig. 1:**
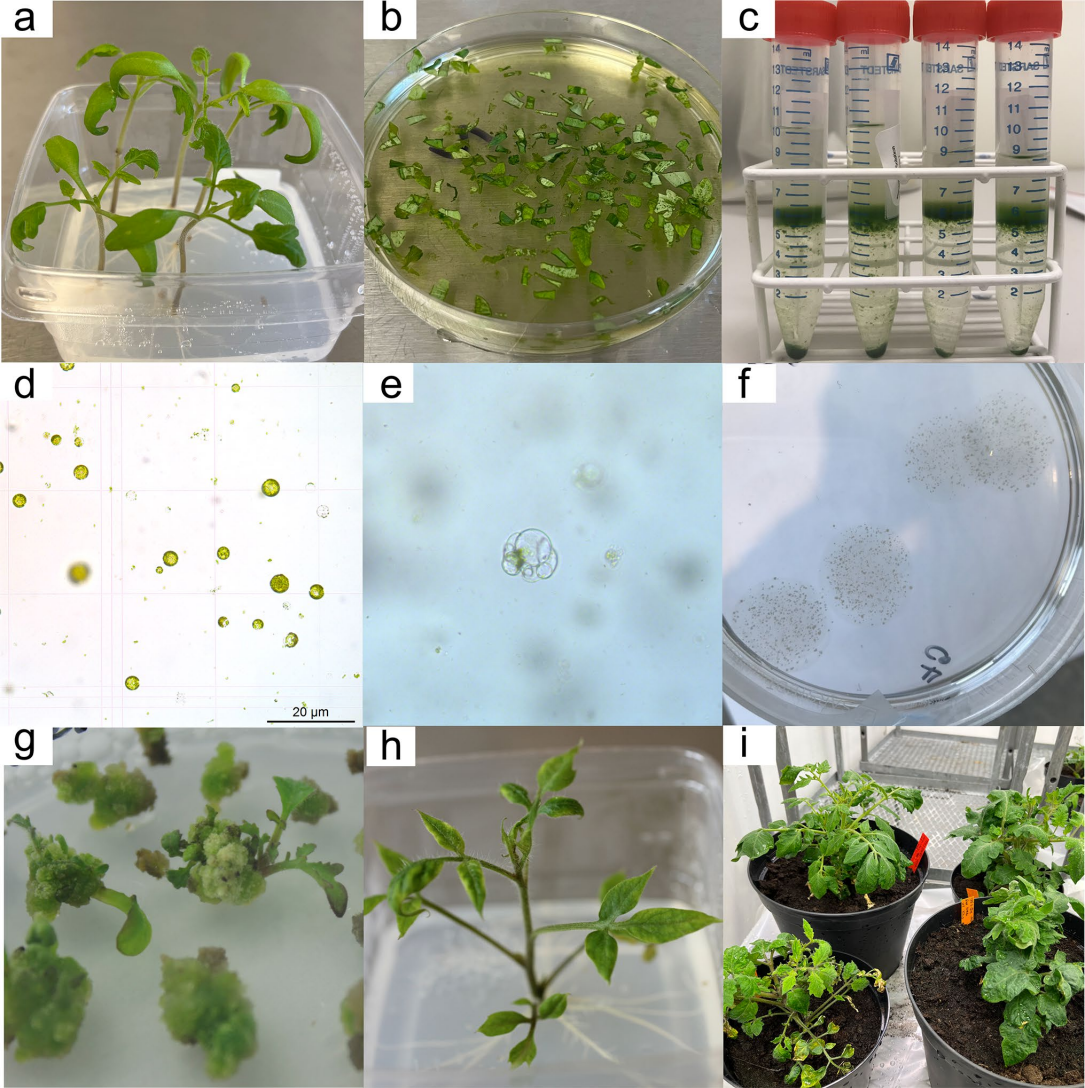
Protoplast isolation and regeneration from tomato (*S. lycopersicum*) cv. Red Setter. **a** Cotyledons and first true leaves from 21-d-old in vitro seedlings used for protoplast isolation. **b** Sliced cotyledons and first true leaves incubated in enzyme solution after 16 h under 25 °C before protoplast purification. **c** Dark green bands containing released intact protoplasts appeared at the interface of sucrose solution and wash solution after centrifugation. **d** Freshly isolated green protoplasts under microscope. **e** Cell division 5 d after protoplast isolation. **f** Callus formation derived from protoplasts embedded in alginate after 12 d from protoplast isolation. **g** Calli released from alginate and cultured on solid shoot regeneration Medium TSR-b with first regenerated shoots observed three months after protoplast isolation. **h** A regenerated plant with well-developed roots on root regeneration medium three months after protoplast isolation. **i** Regenerated plants moved to soil in biotron four months after protoplast isolation

**Fig. 2:**
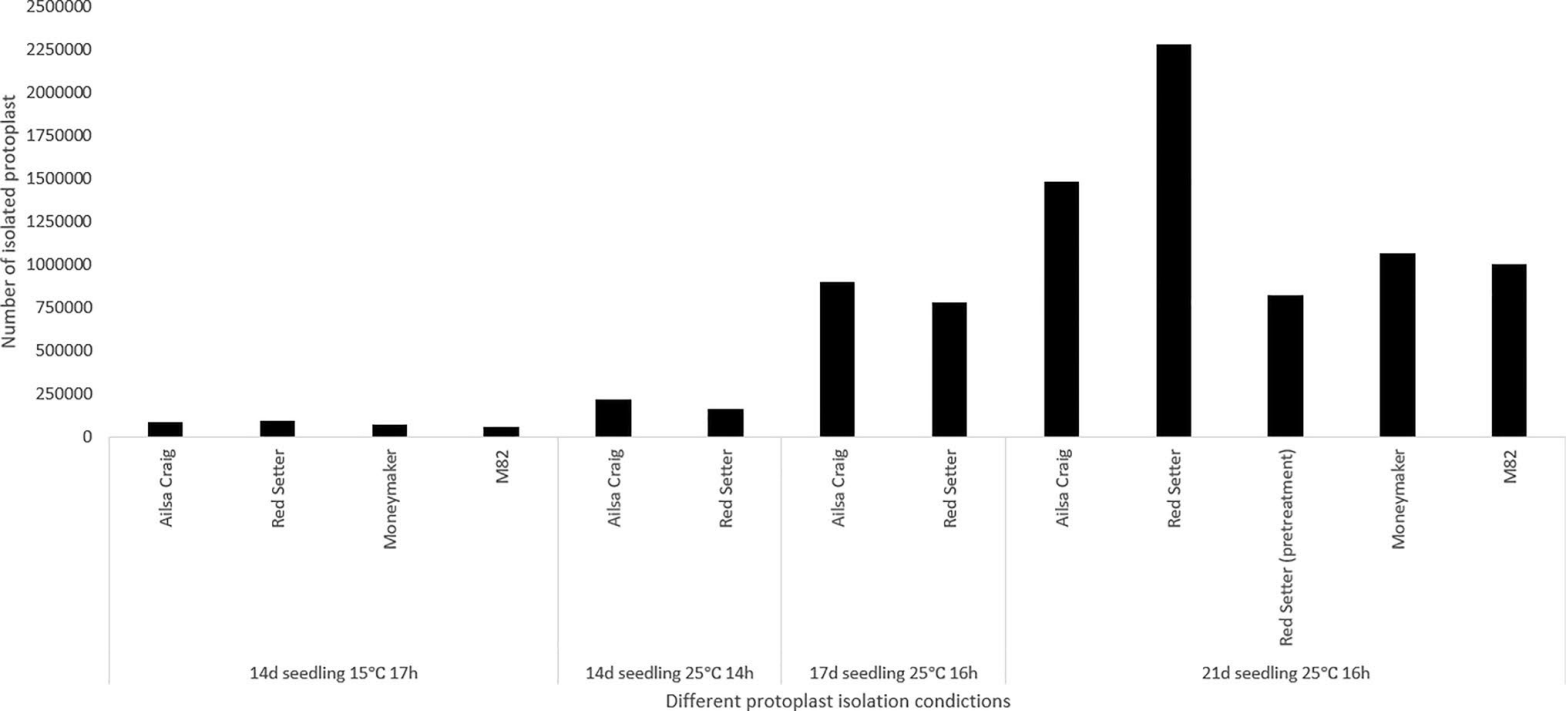
Comparison of effects of different protoplast isolation conditions on the protoplast yield from four different tomato cultivars (Red Setter, Ailsa Craig, M82 and Moneymaker). The number of isolated protoplasts was calculated from the extraction and sampling of 1 g seedlings (results are normalized)

To improve yield, viability and regenerability of isolated protoplasts, the process was optimized in this study based on a previously published method (Nicolia et al. 2021a). The key steps of protoplast isolation are shown in Fig. 1a–d. The yield of protoplasts with initial isolation conditions (14 d seedlings, enzyme treatment: 15 °C for 17 h) from the four cultivars is shown in Fig. 2 and the optimization efforts were made on two cvs, Red Setter and Ailsa Craig, using variables, such as different seedling age, enzyme digestion temperature and duration, and preconditioning treatment.

We found that the yield of extracted protoplasts was improved using older seedlings, higher enzyme treatment temperature and longer incubation time. The number of isolated protoplasts from seedlings at the age of 21 d with the enzyme digestion at 25 °C for 16 h was 15–25 times higher than when using 14-d-old seedlings with the enzyme treatment at 15 °C for 17 h in all four tested cultivars, where a thick dark green band was formed after purification using sucrose density gradient centrifugation (Fig. 1c). An extra pretreatment step of in vitro seedlings (cv. Red Setter) before protoplast isolation did not increase the yield of protoplasts, as shown in Fig. 2.

To confirm our findings for optimized protoplast isolation, we tested two additional tomato cvs, M82 and Moneymaker, under the optimized protoplast isolation conditions. The results illustrated that the yield of protoplasts obtained from cvs M82 and Moneymaker was also improved using the new protoplast isolation conditions.

### Cell division and callus formation from RNP-transfected protoplasts (week 1–4)

Freshly isolated protoplasts (Fig. 1d) were used for PEG transfection with RNP complexes or a vector harboring GFP. Expression of GFP was observed under microscope after 24 h incubation at room temperature and the estimated transfection efficiency was 30–50% (Supp. Figure 1a). RNP-transfected protoplasts were embedded in alginate and incubated at 25 °C. After 4–5-d incubation in the dark, initial cell division was observed under microscope (Fig. 1e). Light was gradually increased, and the mini-calli were usually visible to the naked eye 2 weeks after transfection (Fig. 1f).

### Shoot regeneration on different solid media (week 4–10) and root formation (week 9–12)

To find the optimal solid tomato shoot regeneration (TSR) medium, different media compositions were assessed. Calli were released from alginate when the size reached 1–3 mm (usually 5 weeks after transfection) and moved to the various solid media for assessment of shoot regeneration. Media were designed to study the effect of different combinations or concentrations of plant hormones, different gelling agents and different carbon sources on shoot regeneration. The results of shoot regeneration rate from non-treated and treated protoplasts of the cv. Red Setter on three different shoot regeneration media, are summarized in Table 1 (for results with all tested media see Supp. Table 3). Shoot primordia were observed 2–12 weeks after moving to different shoot regeneration media. The highest shoot regeneration rate (from RNP-transfected protoplasts) was on Medium TSR-a (31.4%) and Medium TSR-b (21.3%), without significant difference. While there was no significant difference among the three treatments (*p* = 0.844), differences for the various media (*p* = 0.007) and the interaction between treatments and media were both significant (*p* < 0.001).

**Table 1:** Shoot regeneration rate^1^ (%) on three media, TSR a-c (cv. Red Setter)

Treatment	Regeneration rate (%) on TSR Media^2^		
	TSR-a	TSR-b	TSR-c
Protoplasts + PEG + RNPs	31.4 a	21.3ab	18.4b
Protoplasts + PEG	19.3a	30.3a	25.4a
Protoplasts	8.1b	44.6a	24.5ab

^1^The calculation of regeneration rate is described in detail in material and methods. Values in a row followed by the same letters were not statistically different at *p* = 0.05 (*n* = 3)

^2^TSR Medium a, b and c are different media for shoot regeneration and the components of each medium are shown in Supp. Table 3

When the shoots reached a length of 1–2 cm and at least two leaves had developed, they were excised from the calli (the calli was discarded after picking one shoot) and transferred to root regeneration medium. Usually well-developed roots were formed within two weeks.

Interestingly, there was morphological difference among shoots regenerated on different shoot regeneration media using cultivar Red Setter (Supp. Figure 1), which had an effect on root formation. For example, the shoots from Medium TSR-b (Fig. 1g) were green and healthy without any evident defect and usually produced well and fast developed roots (Fig. 1h) with normal rooting and acclimation in pots (Fig. 1i). On the other hand, the shoots regenerated from Medium TSR-a were curved, less green, with a grass-like shape (Supp. Figure 1b). When moved to root regeneration medium, roots developed more slowly and shoots even failed to survive. Contrasting with this, the shoots on Medium TSR-c (Supp. Figure 1c) were thicker, and faced the same rooting issue as using Medium TSR-a.

### *SP* and *SP5G* allele sequencing and sgRNA design

**Fig. 3:**
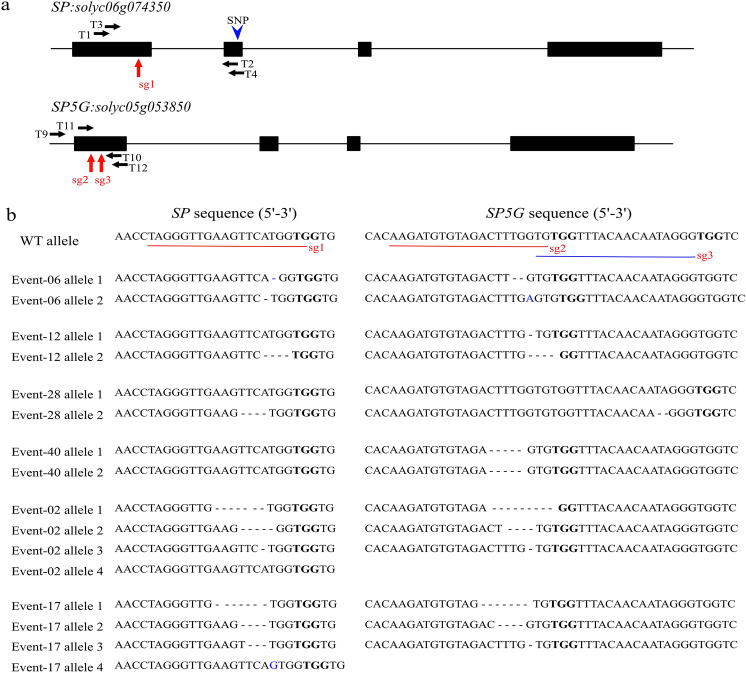
DNA-free CRISPR/Cas9 mediated genome editing in tomato multiplexing of *SP* and *SP5G* genes. **a** Structure of *SP* and *SP5G* genes. Exons are indicated in black boxes. Primers used for genotyping and sequencing are noted with black arrows. For each gene, sgRNAs (red arrows) were designed, all targeting exon 1. Only one SNP (blue arrow) was found within the amplification region of the *SP* gene. **b** Genotyping of first-generation events (M_0_) by Sanger Sequencing. The DNA sequence of each allele was aligned to wild type (WT) allele and deletions are shown with hyphens and insertions marked with blue color. Protospacer Adjacent Motif (PAM) is shown in bold

For the determination of *SP* and *SP5G* allele gene sequences, we designed two different primer pairs for PCR amplification covering the exon 1 region of all four used cultivars (Fig. 3a). Sequence results showed that in the amplified region, they were identical to the public tomato reference genomic sequences, except for the *SP* gene, where one single-nucleotide polymorphism (SNP) was identified among the four cultivars (Supp. Figure 2).

One sgRNA (sgRNA1) for *SP* and two sgRNAs for *SP5G* (sgRNA2 and sgRNA3) were designed (Fig. 3a) and used for multiplexing of the targets in two different combinations, sgRNA1 + 2 and sgRNA1 + 3.

### Identification of mutants after multiplexed targeting of *SP* and *SP5G*

In total we analyzed 110 regenerated shoots (events, M_0_ plants) by HRFA analysis (for HRFA results on all mutants see Supp. Table 4), where *SP* and *SP5G* genes were targeted simultaneously by either sgRNA1 + 2 or sgRNA1 + 3. Among all 110 events, 66 (60.0%) were identified with mutations (indels found in at least one allele) in either *SP* or *SP5G* genes (Table 2). Of mutated events, 10 (9.1%) and 21 (19.1%) events were edited only in *SP* and *SP5G*, respectively, while the remaining were mutated in both genes. Furthermore, 34 (30.9%) events were found to be potentially chimeric from the observation that more than two allelic variants of either gene were detected during HRFA analysis. We selected 20 events for genotyping by Sanger Sequencing and the results were in line with the indel sizes identified with HRFA analysis, as can be seen in Fig. 3b and Supp. Table 4. We selected 14 representative regenerated M_0_ plants and five unedited regenerated plants and grew them in the biotron for further phenotypical assessment (Supp. Figure 3).

**Table 2:** Mutation rate^1^ of regenerated events (M_0_) from transfected protoplasts, tomato cv. Red Setter

total # of events analyzed	# of events with mutation^2^	# of events with mutation only in *SP*	# of events with mutation only in *SP5G*	# of events with mutation in both SP and *SP5G*	# of events possibly chimeric^3^
110	66 (60.0%)	10 (9.1%)	21 (19.1%)	35 (31.8%)	34 (30.9%)

^1^Mutations (indels) were determined on a single leaf from 110 regenerated plants by HRFA analysis where both *SP* and *SP5G* genes were targeted simultaneously

^2^Mutations in at least one allele in either *SP* or *SP5G* genes. The results of HRFA analysis of all 66 mutated events are shown in Supp. Table 4

^3^More than two allelic variants for either *SP* or *SP5G* detected in an event

## Discussion

Genome editing has become a complementary method to traditional breeding of many crops including tomato and the CRISPR/Cas9 technology is the most utilized tool in recent years. Tomato, as an important horticultural crop with high commercial value, has already been well studied genetically, which makes the application of modern molecular breeding possible (Foolad 2007). Currently, there is no DNA-free genome editing method established for cultivated tomato, which is an important drawback when utilizing this important technology for breeding or in research. A DNA-free genome editing method requires efficient and reproducible shoot regeneration from single cells, which is still a challenge and can be highly genotype dependent (Peres et al. 2001). A recent study reported protoplast regeneration via a DNA-free method on wild tomato, which is the closest study so far to cultivated tomato (Lin et al. 2022). Based on a previously published protocol for cultivated tomato protoplast genome editing via RNP-based CRISPR/Cas9 (Nicolia et al. 2021a), we have improved the protoplast isolation process and solved the challenge of shoot regeneration from RNP-transfected protoplasts.

The yield and quality of isolated protoplasts further affect shoot regeneration. Here we have optimized the process of protoplast isolation based on seedling age and enzyme digestion temperature and duration. With the optimized conditions (21 d seedlings, enzyme treatment: 25 °C for 16 h), we successfully increased the yield of protoplasts to 1.0–2.2 × 10^6^ per gram of leaf materials, which were comparable results to previous reports on tomato (Morgan and Cocking 1982; Niedz et al. 1985; Tan et al. 1987). We also found in this study that preconditioning treatment of donor plants under 4 °C prior to protoplast isolation had a negative effect on protoplast yield. By contrast, Tan et al. (1987) got the opposite result from preconditioning treatment, where they found that cold treatment increased the stability of protoplasts and thus yielded more viable protoplasts.

The formation of callus from transfected protoplasts is achieved by stimulation of cell wall development and cell divisions. Moreover, there are many factors that can affect the success of shoot regeneration, such as osmotic pressure, different types and concentration of hormones, carbon sources and gelling reagents. In this study, shoot regeneration was analyzed on ten different shoot regeneration media, but with extra focus on three of them. Cytokinins, such as zeatin and 6-Benzylaminopurine (6-BAP), are involved in early cell division as well as initiation and elongation of shoots, and are widely used in protoplast-derived shoot regeneration in many plant species. Previous studies showed that zeatin was necessary for tomato shoot regeneration (Morgan and Cocking 1982) and we found that the 0.1 mg/L IAA and 0.75 mg/L zeatin was the most suitable combination for shoot regeneration in all ten media tested with cv. Red Setter. On the other hand, when the calli were cultured on 6-BAP-based media together with IAA or 1-Naphthaleneacetic acid (NAA) (Medium TSR-d and TSR-e) (Supp. Figure 1d, e), the browning of calli seemed to accelerate or smaller calli were generated and no shoots were regenerated after six months. Gibberellins such as Gibberellic acid (GA_3_) have been proven to be beneficial for stimulating shoot elongation (Niedz et al. 1985). Shahin (1985) observed higher regeneration rate when using both zeatin and GA_3_ compared with using zeatin alone. In contrast, we found that when GA_3_ was added in early shoot induction process, it had an adverse effect on shoot morphology which was curved, thin and grass-like as observed from most of the shoots regenerated from Medium TSR-a, TSR-f and TSR-g (Supp. Figure 1b, f and g) where GA_3_ concentration varied from 0.34 mg/L to 1 mg/L. Auxins are also an essential component in shoot regeneration medium, such as the frequently used IAA and NAA. We found that when IAA was replaced by NAA, the calli on Medium TSR-h were inflated, less green and not able to generate shoots (Supp Fig. 1h), which did not concur with the conclusions from Niedz et al. (1985). We did not find an obvious difference between two carbon sources and gelling agent in this study.

In this study, four different tomato cvs Red Setter, Ailsa Craig, M82 and Moneymaker were used to study protoplast regeneration from RNP-transfected protoplasts. Cultivar Red Setter was superior to other cvs with a regeneration rate up to 31.4% from RNP-transfected protoplasts. Five shoots were obtained from 200 RNP-transfected protoplast-derived calli from cv. M82, with a high mutation rate (80%), although all four mutant regenerated events were chimeras (Supp. Figure 4). On the contrary, plating efficiency was low on both cvs Ailsa Craig and Moneymaker and all attempts to regenerate shoots from RNP-transfected protoplasts failed, indicating that a further adaptation of the protocol will be required for these cultivars. In this study, we clearly observed genotype differences among the four tested cultivars, which was in line with previous reports where variable regeneration rate among cultivars was found (Morgan and Cocking 1982; Niedz et al. 1985; Tan et al. 1987).

The mutations identified in M_0_ events were a mix of bi-allelic, mono-allelic and chimeric with small deletions or insertions at the target site with an editing efficiency up to 60% in considering at least one allele mutated in either *SP* or *SP5G* genes and 31.8% considering on both genes simultaneously targeted. Previously, 30 and 90% of protoplast-derived calli were found to be mutated in at least one allele of *CCD7* or *CCD8* genes, respectively, after multiplex RNP delivery (Nicolia et al. 2021a). Such results indicate that the CRISPR/Cas9 system using RNP-transfected protoplasts can be highly efficient to generate desired mutations in cultivated tomato, without any stable integration of foreign DNA. Brooks et al. (2014) were the first to report the successful application of the CRISPR/Cas9 system on tomato via stable *Agrobacterium tumefaciens*-mediated transformation with an editing efficiency of 48% on T_0_ plants with two sgRNAs targeting at the same gene. More studies using CRISPR/Cas9 via *Agrobacterium tumefaciens*-mediated transformation have been published in recent years and some of these studies had a very high editing efficiency of up to 100% of the transgenic shoots (Ito et al. 2015; Ueta et al. 2017; Dahan-Meir et al. 2018). With the latter method, however, comes the use of antibiotic selection as well as either selfing or backcrossing to remove T-DNA insertions if a transgene-free plant is desirable.

We observed a higher rate of potential chimeras in tomato than for example, potato, using RNP complexes and similar protoplast density (Andersson et al. 2018). This might be because the ratio between RNP complexes and protoplasts was not optimal in our study, as Sidorov et al. (2021) also observed high number of chimeras (33.3%) on regenerated calli from RNP-transfected protoplasts in canola (*Brassica napus* L.). It might be possible to address it by testing higher concentrations of RNPs as the efficiency of RNP is dose dependent (Zhang et al. 2022). Another possible reason might be endopolyploidy according to a report by Smulders et al. (1994) on varying ploidy level in different tomato somatic tissues. A high chimeras using CRISPR/Cas9 on tomato was also identified in earlier studies on *Agrobacterium tumefaciens*-mediated transformation (Brooks et al. 2014; Dahan-Meir et al. 2018). However, a high frequency of chimeric events is less important in tomato than in clonally propagated crops, due to sexual generation and the possibility of selecting and producing homozygous mutations in the next generation. Moreover, the use of protoplasts allows to scale-up the experiments of mutagenesis easily, so that among a high number of regenerated mutant M_0_ plants those indicating chimerism can be discarded.

Our findings illustrate that the challenge of shoot regeneration from transfected protoplasts of cultivated tomato has been overcome and we have successfully obtained regenerated plants from non-treated protoplasts from all four studied cultivars, as well as regenerated plants with induced mutations in two cultivars (Red Setter and M82) via DNA-free CRISPR/Cas9. The availability of this reported method in the determinate cvs Red Setter or M82 provides opportunities for important research and breeding efforts oriented toward tomato field cultivation and industrial processing, such as improving the fruit quality (e.g., flavor, sugar content, acidity) and plant resistance to biotic (e.g., soil born, virus, parasitic plants) and abiotic stress (e.g., water deficiency, salinity).

## Supplementary Information

Below is the link to the electronic supplementary material.

Supplementary file1 (DOCX 8245 KB)

## Data Availability

All the data in this study are included in this manuscript and supplementary data file.
