# Supplementary material for: Establishment of a DNA-free genome editing and protoplast regeneration method in cultivated tomato (Solanum lycopersicum)
Source: Plant Cell Rep. 2022 Jun 30;41(9):1843–52. doi: 10.1007/s00299-022-02893-8 (PMC9395478; doi:10.1007/s00299-022-02893-8)
Supplement: Supplementary file 1 — Supplementary file1 (DOCX 8245 KB) [file 299_2022_2893_MOESM1_ESM.docx]

**Supplementary Data**

**Establishment of a DNA-free genome editing and protoplast regeneration method in cultivated tomato (*Solanum lycopersicum*)**

**Ying Liu^1^*, Mariette Andersson^1^, Antonio Granell^2^, Teodoro Cardi^3,4^, Per Hofvander^1^, Alessandro Nicolia^3^**

* corresponding author

^1^ Department of Plant Breeding, Swedish University of Agricultural Sciences, P.O. Box 190, 23422 Lomma, Sweden

^2^ Instituto de Biología Molecular y Celular de Plantas, CSIC-Universidad Politécnica de Valencia, Valencia 46022, Spain

^3^ Council for Agricultural Research and Economics, Research Centre for Vegetable and Ornamental Crops, Via Cavalleggeri 25, 84098 Pontecagnano, Italy

^4^ CNR-IBBR , Institute of Biosciences and Bioresources, via Università 133, 80055 Portici, Italy

**Contents**

**Supplementary Figure 1.** GFP expression and different morphologic shoots regenerated from various media (cv. Red Setter)

**Supplementary Figure 2.** Nucleotide sequence of *SP* and *SP5G* genes (cv. Red Setter)

**Supplementary Figure 3.** Phenotype of representative regenerated M_0_ plants (cv. Red Setter)

**Supplementary Figure 4.** Representative mutations in M_0_ regenerated plants (cv. M82)

**Supplementary Table 1.** Compounds of different TSR media

**Supplementary Table 2.** Primers and sgRNAs used in this study

**Supplementary Table 3.** Regeneration rate on other shoot regeneration media (cv. Red Setter)

**Supplementary Table 4.** Mutants in regenerated M_0_ plants (cv. Red Setter) analyzed by HRFA analysis

**Supplementary Figure 1.** GFP expression and different morphologic shoots regenerated from various media (cv. Red Setter). **a** GFP expression in transfected protoplast 24 h after incubation. **b** Shoots regenerated on TSR-a. **c** Shoots regenerated on Medium TSR-c. **d** Calli incubated on Medium TSR-d. **e** Calli incubated on TSR-e. **f** Shoots regenerated on Medium TSR-f. **g** Calli incubated on Medium TSR-g. **h** Calli incubated on Medium TSR-h. **i** Shoots regenerated on Medium TSR-i. **j** Shoots regenerated on Medium TSR-j. **k** Shoot regenerated from only GFP-transfected protoplasts from cv. Moneymaker on Medium TSR-b. **l** Shoot regenerated from only GFP-transfected protoplasts from cv. Ailsa Craig on Medium TSR-b.


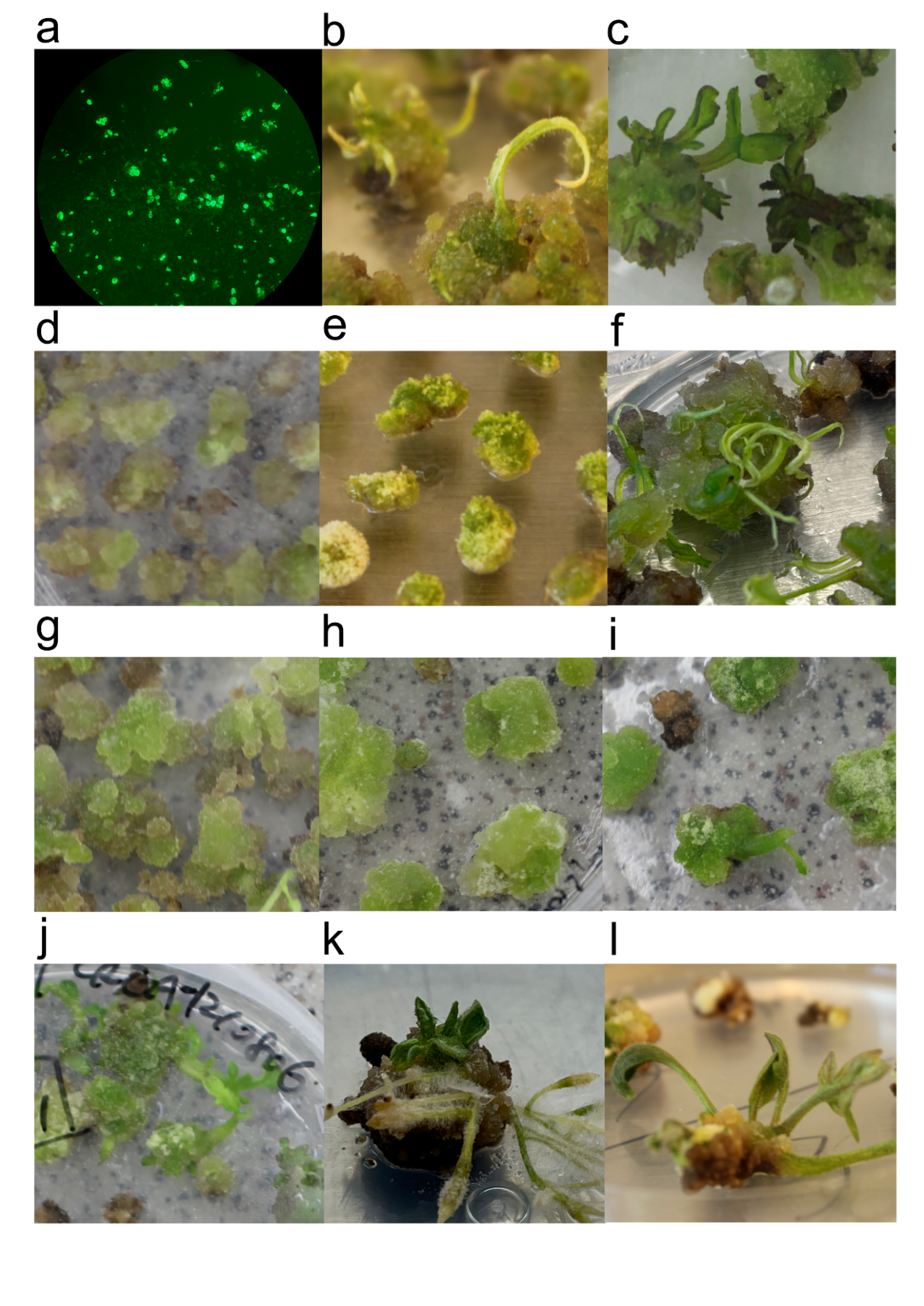


**Supplementary Figure 2.** Nucleotide sequence of *SP* and *SP5G* genes (cv. Red Setter). **a** Nucleotide sequence of *SP* gene and only one SNP identified within the amplified region among four different cultivars as noted with blue arrow: it is "T" in cv. Red Setter and M82, while in cv. Ailsa Craig and Moneymaker is "C". **b** Nucleotide sequence of *SP5G* gene and no SNP was found within the amplified region. The nucleotide sequence of sgRNAs used in this study was indicated with light green arrows as sg1, sg2 and sg3, respectively. The primers are shown in red arrows as T3, T4, T11 and T12, respectively.


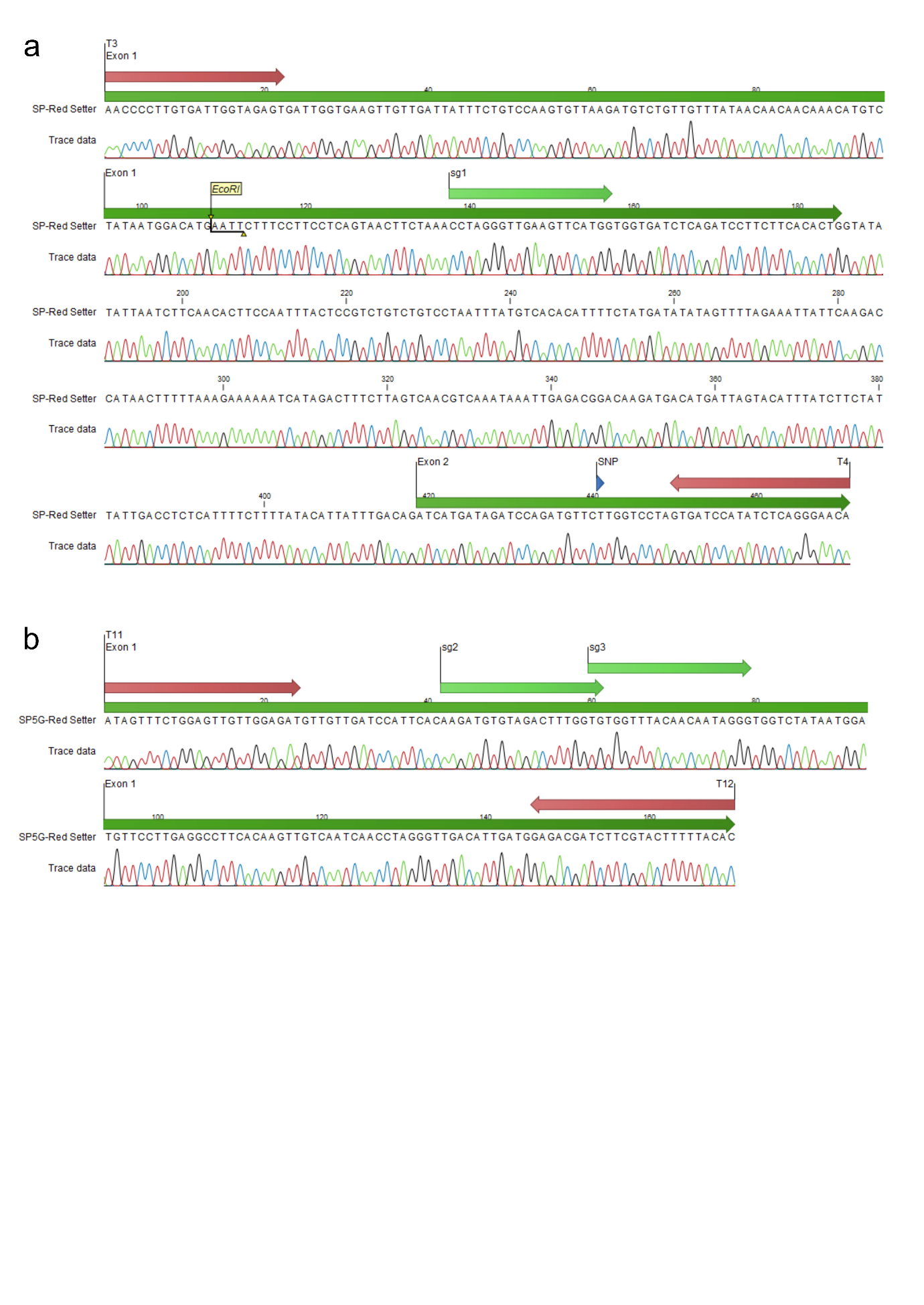


**Supplementary Figure 3.** Phenotype of representative regenerated M_0_ plants (cv. Red Setter).


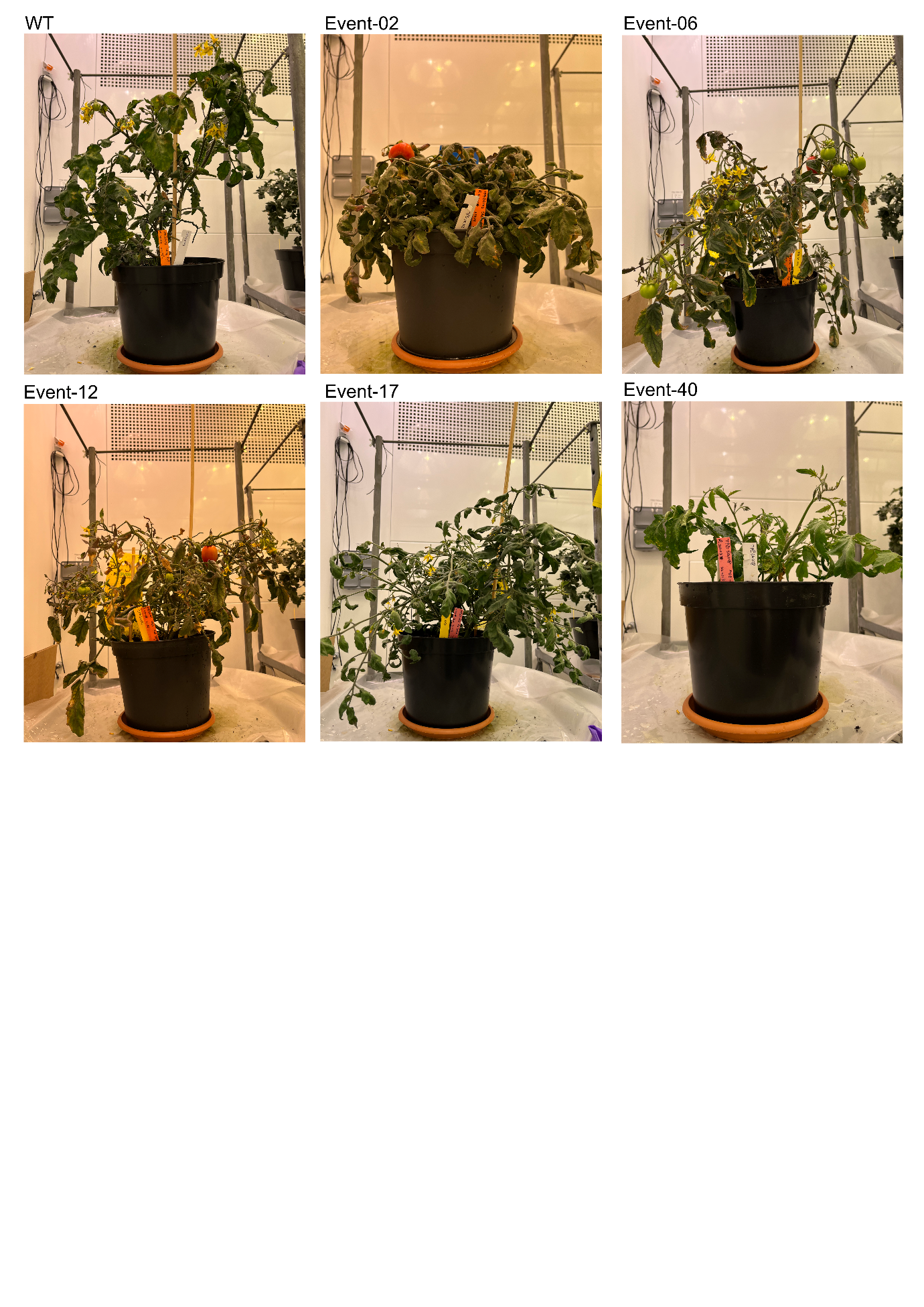


**Supplementary Figure 4.** Representative mutations in M_0_ regenerated plants (cv. M82). **a** Genotyping of first-generation events (M_0_) by Sanger Sequencing. **b** Phenotype of two M_0_ lines in biotron.


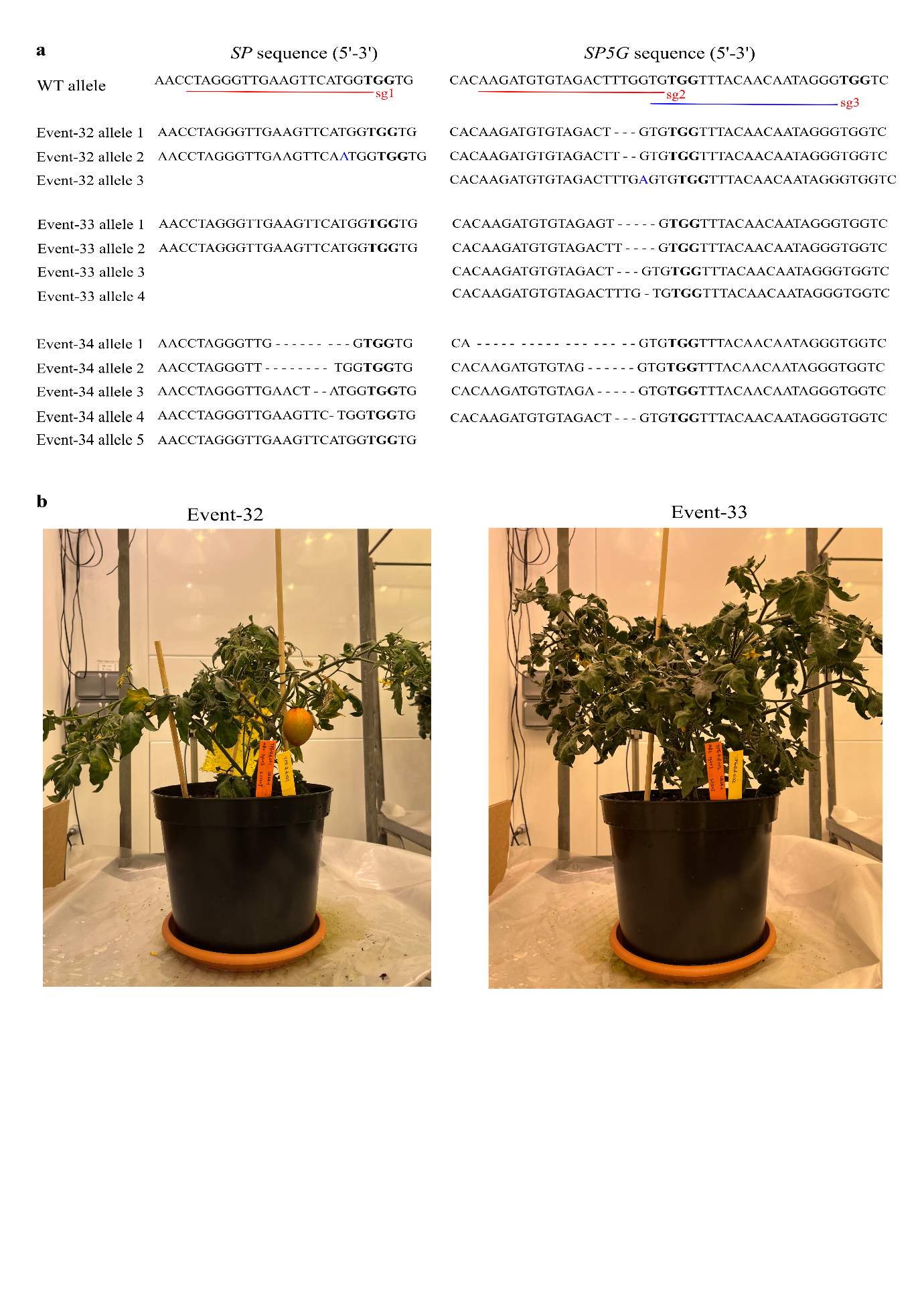


**Supplementary Table 1.** Compounds of different TSR media.

| **Medium^1^** | **MS salt^2^** | **MS salt^3^** | **MS salt^4^** | **Glucose** | **Sucrose** | **Mannitol** | **IAA** | **Zeatin** | **BAP** | **NAA** | **GA3** | **Phyto agar** | **Gelrite** | **Reference^5^** |
| --- | --- | --- | --- | --- | --- | --- | --- | --- | --- | --- | --- | --- | --- | --- |
|  | **g/L** | **g/L** | **g/L** | **g/L** | **g/L** | **g/L** | **mg/L** | **mg/L** | **mg/L** | **mg/L** | **mg/L** | **g/L** | **g/L** |  |
| TSR-a |  |  | 4.405 |  | 10 |  |  | 2 |  | 0.01 | 0.1 |  | 2.5 | Nicolia et al. 2015 |
| TSR-b | 4.9 |  |  | 20 |  |  | 0.1 | 0.75 |  |  |  | 8 |  | Antonio Granell |
| TSR-c |  | 4.302 |  |  |  |  |  | 2 |  |  |  |  | 2.5 | Morgan and Cocking 1982 |
| TSR-d | 4.9 |  |  |  | 20 |  | 1.75 |  | 2.25 |  |  | 8 |  | Mühlbach 1980 |
| TSR-e |  | 4.302 |  |  | 2.5 | 36.4 |  |  | 0.5 | 0.05 |  |  | 2.5 | Tan et al. 1987 |
| TSR-f | 4.302 |  |  |  | 20 |  |  | 1 |  |  | 0.2 |  | 2.5 | Shahin 1985 |
| TSR-g | 4.302 |  |  |  | 20 |  |  | 0.88 |  |  | 0.34 | 8 |  | Hossain et al. 1995 |
| TSR-h |  | 4.302 |  |  | 20 |  |  | 2 |  | 0.5 |  |  | 2.5 | Li et al. 2021 |
| TSR-i |  | 4.302 |  |  | 20 |  |  | 2 |  |  |  |  | 2.5 | Morgan and Cocking 1982 |
| TSR-j | 4.9 |  |  |  | 20 |  | 0.02 | 2 |  |  |  | 8 |  | Tan et al. 1987 |

^1^TSR medium a-j are different tomato shoot regeneration media used in this study.

^2^MS salt (1962) including vitamins and MES (Duchefa M0255)

^3^MS salt including vitamins (Duchefa M0222)

^4^MS salt (Duchefa M0221)

^5^Media was designed and modified based on different reference.

**Supplementary Table 2.** Primers and sgRNAs used in this study

| **Name** | **Sequence (5'-3')** |
| --- | --- |
| **Primers** | |
| T1 | GCTTCCAAAATGTGTGAACCC |
| T2 | ATCACTAGGACCAAGAACATCTGG |
| T3* | AACCCCTTGTGATTGGTAGAGT |
| T4* | TGTTCCCTGAGATATGGATCAC |
| T9 | TTCACGACTTGTCAACCATTG |
| T10 | ACGAAGATCGTCTCCATCAA |
| T11* | ATAGTTTCTGGAGTTGTTGGAGAT |
| T12* | GTGTAAAAAGTACGAAGATCGTCTC |
| **sgRNAs** | |
| sg1 | CCTAGGGTTGAAGTTCATGG |
| sg2 | AAGATGTGTAGACTTTGGTG |
| sg3 | TGTGGTTTACAACAATAGGG |

* Analytical primers used for HRFA analysis (together with FAM/Hex fluorescent dye).

**Supplementary Table 3.** Regeneration rate^1^ on other shoot regeneration media (cv. Red Setter)

| **Treatment** | **# of regenerated shoots (regeneration rate) on TSR Media** | | | | | | |
| --- | --- | --- | --- | --- | --- | --- | --- |
|  | **TSR-f** | **TSR-j** | **TSR-g** | **TSR-i** | **TSR-d** | **TSR-h** | **TSR-e** |
| Protoplasts + PEG + RNPs | 15 (30.0%) | 10 (20.0%) | 9 (18.0%) | 5 (10%) | 0 | 0 | 0 |
| Protoplasts + PEG | 14 (28.0%) | 19 (38.0%) | 7 (14%) | 20 (40%) | 0 | 0 | 0 |

^1^Regeneration rate was calculated based on one replicate (each replicate included 50 callus).

**Supplementary Table 4.** Mutants^1^ in regenerated M_0_ plants (cv. Red Setter) analyzed by HRFA analysis.

| **M_0_ plants** | ***SP*** | | | | | ***SP5G*** | | | |
| --- | --- | --- | --- | --- | --- | --- | --- | --- | --- |
|  | **allele 1** | **allele 2** | **allele 3** | **allele 4** | **allele 5** | **allele 1** | **allele 2** | **allele 3** | **allele 4** |
| Event-01 | -36 | -2 | 0 | 1 |  | -2 | -3 | 1 |  |
| Event-02* | -7 | -5 | -1 | 0 |  | -9 | -4 | -1 |  |
| Event-05 | -1 | 0 |  |  |  | -6 | -5 | -4 |  |
| Event-06* | -1 | -1 |  |  |  | -2 | 1 |  |  |
| Event-07 | 0 | 1 |  |  |  | 0 | 0 |  |  |
| Event-10* | -4 | 0 |  |  |  | 0 | 0 |  |  |
| Event-11 | -1 | 0 |  |  |  | -3 | -2 | -1 | 0 |
| Event-12* | -4 | 0 |  |  |  | -4 | -1 |  |  |
| Event-13 | 0 | 0 |  |  |  | -1 | 0 | 1 |  |
| Event-15 | 0 | 1 |  |  |  | -76 | -4 | -1 | 0 |
| Event-16 | 0 | 0 |  |  |  | -5 | 0 | 1 |  |
| Event-17* | -7 | -4 | -3 | 1 |  | -7 | -4 | -1 |  |
| Event-18 | -7 | -5 | -1 | 0 |  | -9 | -4 | -1 |  |
| Event-19 | 0 | 1 |  |  |  | 0 | 0 |  |  |
| Event-20 | -5 | -1 | 0 | 1 |  | -5 | -3 |  |  |
| Event-22* | 0 | 1 |  |  |  | 0 | 0 |  |  |
| Event-23 | 0 | 1 |  |  |  | 0 | 0 |  |  |
| Event-24 | -2 | 0 |  |  |  | -6 | -4 | -3 | 1 |
| Event-25 | -5 | 0 |  |  |  | 0 |  |  |  |
| Event-26 | -5 | 0 |  |  |  | -5 | -3 | -2 | 0 |
| Event-27 | 0 | 0 |  |  |  | 0 | 1 |  |  |
| Event-28* | -4 | 0 |  |  |  | -2 | 0 |  |  |
| Event-29 | 0 | 1 |  |  |  | 0 | 0 |  |  |
| Event-30 | 0 | 0 |  |  |  | -5 | 0 |  |  |
| Event-35 | 0 | 0 |  |  |  | -1 | 0 |  |  |
| Event-37 | -9 | -6 | -2 | 0 |  | -14 | -4 | -1 |  |
| Event-38 | 0 | 1 |  |  |  | -1 | 0 |  |  |
| Event-40* | 0 | 0 |  |  |  | -5 | -5 |  |  |
| Event-43* | 0 | 0 |  |  |  | -5 | -4 | 0 |  |
| Event-45 | 0 | 0 |  |  |  | -1 | 0 |  |  |
| Event-47 | -6 | -5 | -1 |  |  | -12 | -6 | -3 |  |
| Event-48 | 0 | 0 |  |  |  | -3 | 0 |  |  |
| Event-49 | -2 | 0 |  |  |  | -6 | -4 | 0 |  |
| Event-52* | 0 | 0 |  |  |  | -4 | 0 |  |  |
| Event-55 | 0 | 0 |  |  |  | -3 | -2 | 0 |  |
| Event-56* | -1 | 0 |  |  |  | -1 | 0 |  |  |
| Event-59* | 0 | 0 |  |  |  | -7 | -1 | 0 |  |
| Event-64 | 0 | 0 |  |  |  | -7 | -1 | 0 |  |
| Event-65 | -1 | 0 |  |  |  | -12 | -4 | -1 | 0 |
| Event-66 | 0 | 1 |  |  |  | -4 | 0 |  |  |
| Event-67 | 0 | 1 |  |  |  | -3 | 0 |  |  |
| Event-69 | -8 | 0 |  |  |  | -1 | 0 | 1 |  |
| Event-70 | 0 | 0 |  |  |  | 0 | 1 |  |  |
| Event-75 | -1 | 0 |  |  |  | -4 | 0 | 1 |  |
| Event-77 | -11 | -4 | -1 | 0 | 1 | -2 | -1 | 0 | 1 |
| Event-79* | -2 | 0 |  |  |  | -2 | 0 |  |  |
| Event-81 | -7 | -3 | 0 |  |  | 0 | 1 |  |  |
| Event-84 | -1 | 0 |  |  |  | -1 | 0 |  |  |
| Event-85* | -143 | 0 |  |  |  | -2 | -1 | 1 |  |
| Event-89 | -2 | 0 |  |  |  | -6 | -4 | -3 | 1 |
| Event-91 | -3 | 0 |  |  |  | 0 | 0 |  |  |
| Event-92 | -3 | 0 |  |  |  | 0 | 0 |  |  |
| Event-93 | 0 | 0 |  |  |  | 0 | 1 |  |  |
| Event-95 | -143 | -4 | -2 | 0 |  | -2 | -1 | 0 | 1 |
| Event-97 | 0 | 0 |  |  |  | 0 | 1 |  |  |
| Event-98 | 0 | 0 |  |  |  | 0 | 1 |  |  |
| Event-99 | 0 | 0 |  |  |  | 0 | 1 |  |  |
| Event-100 | 0 | 0 |  |  |  | 0 | 1 |  |  |
| Event-101 | -3 | 0 |  |  |  | -6 | -5 | -2 | 0 |
| Event-104 | 0 | 0 |  |  |  | -5 | -4 | 0 |  |
| Event-105 | -1 | 0 | 1 |  |  | -5 | -4 | -1 | 1 |
| Event-106 | -1 | 0 |  |  |  | -6 | -4 | -1 |  |
| Event-109 | -9 | 0 | 1 |  |  | -6 | -4 | -2 |  |
| Event-112 | 0 | 0 |  |  |  | -5 | -3 | -2 | 0 |
| Event-113* | -7 | 0 |  |  |  | 0 | 0 |  |  |
| Event-114* | -2 | -1 | 0 |  |  | -4 | -1 | 0 |  |

^1^Mutations were analyzed by HRFA analysis and desired events with “*” were confirmed by Sanger Sequence. Indels with deletion were noted with “-” e.g. “-1” meant 1bp deletion and “1” meant 1bp insertion. WT allele was illustrated as “0”. Mutants with more than 2 alleles (including WT) identified were classified as chimeric mutation.

**Reference**

Hossain M, Imanishi S, Egashira H (1995) An improvement of tomato protoplast culture for rapid plant regeneration. Plant Cell Tissue Organ Cult 42:141–146. https://doi.org/10.1007/BF00034230

Li X, Sandgrind S, Moss O, et al (2021) Efficient Protoplast Regeneration Protocol and CRISPR/Cas9-Mediated Editing of Glucosinolate Transporter (GTR) Genes in Rapeseed (Brassica napus L.). Front Plant Sci 12:1–11. https://doi.org/10.3389/fpls.2021.680859

Morgan A, Cocking EC (1982) Plant Regeneration from Protoplasts of Lycopersicon esculentum Mill. Zeitschrift für Pflanzenphysiologie 106:97–104. https://doi.org/10.1016/s0044-328x(82)80071-8

Mühlbach HP (1980) Different regeneration potential of mesophyll protoplasts from cultivated and a wild species of tomato. Planta 148:89–96. https://doi.org/10.1007/BF00385447

Nicolia A, Proux-Wéra E, Åhman I, et al (2015) Targeted gene mutation in tetraploid potato through transient TALEN expression in protoplasts. J Biotechnol 204:17–24. https://doi.org/10.1016/j.jbiotec.2015.03.021

Shahin EA (1985) Totipotency of tomato protoplasts. Theor Appl Genet 69:235–240. https://doi.org/10.1007/BF00662431

Tan MMC, Rietveld, E.M., et al (1987) Regeneration of leaf mesophyll protoplasts of tomato cultivars (L. esculentum): factors important for efficient protoplast culture and plant regeneration. Plant Cell Rep 6:172–175
